# Good practice: The experiences with the utilization of residual cancer burden—A single institution study

**DOI:** 10.1111/1759-7714.14826

**Published:** 2023-03-03

**Authors:** Anita Sejben, Fanni Hegedűs, Szintia Almási, Márton Berta, Orsolya Oláh‐Németh, Tamás Zombori

**Affiliations:** ^1^ Department of Pathology University of Szeged, Faculty of Medicine Szeged Hungary

**Keywords:** breast cancer, neoadjuvant therapy, reproducibility, residual cancer burden

## Abstract

**Introduction:**

The use of neoadjuvant therapy (NAT) has been showing an incraesing tendency in the treatment of locally advanced breast cancer. The evaluation of residual cancer could be performed by Residual Cancer Burden (RCB) calculator. The prognostic system takes the two largest diameters of the tumor, the cellularity, the amount of in situ carcinoma, the number of metastatic lymph nodes, and the size of the largest metastatic deposit into account. The aim of our study was to examine the reproducibility of RCB in NAT treated patients.

**Methods:**

Patients who were treated with NAT and had resection specimens between 2018 and 2021 were selected. Histological examination was performed by five pathologists. After assessment of the examined variables, RCB points and RCB classes were defined. For statistical analysis, interclass correlation was used (SPSS Statistics V.22.0 software).

**Results:**

Altogether 100 patients were included in our retrospective, cohort study (average age: 57 years). In two‐thirds of the cases, third generation chemotherapy was used, and mastectomy was performed. Significant concordance was found in the two largest diameters of the tumor (coefficients, 0.984 and 0.973), the cellularity (coefficient, 0.970), and the largest metastatic deposit (coefficient, 0.998). Although the amount of in situ carcinoma proved to be the least reproducible factor, it resulted in almost 90% of agreement (coefficient, 0.873). Regarding RCB points and classes, similar results were observed (coefficients, 0.989 and 0.960).

**Conclusions:**

Significant agreement was observed between examiners based on almost all RCB parameters, points, and classes, reflecting the optimal reproducibility of RCB. Therefore, we recommend the use of the calculator in routine histopathological reports in NAT cases.

## Graphical abstract

The evaluation of residual cancer in patients treated with neoadjuvant therapy could be performed by Residual Cancer Burden (RCB) calculator. The prognostic system takes two largest diameters of the tumour, cellularity, amount of in situ carcinoma, number of metastatic lymph nodes, and size of the largest metastatic deposit into account. The aim of our study was to examine the reproducibility of RCB in NAT treated patients. Significant agreement was observed between examiners based on almost all RCB parameters, points and classes, reflecting the optimal reproducibility of RCB. Therefore, we recommend the use of the calculator in routine histopathological reports in NAT cases.

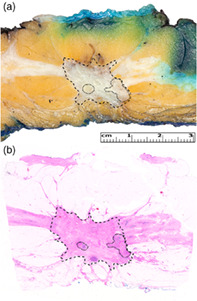

## INTRODUCTION

Neoadjuvant therapy (NAT) has changed the management of breast cancer. NAT is mainly used in cases of locally advanced and/or inoperable breast cancer cases. On variable occasions, tumor regression resulting in downstaging also results in the formerly inoperable patient to be eligible for breast‐conserving surgery. Best prognosis could be achieved in human epidermal growth factor receptor‐2 (HER‐2) positive, and grade 3, triple negative breast cancer (TNBC) cases even at an early stage.^1^, ^2^, ^3^

The outcome is determined by the residual tumor burden in the breast and in the axillary lymph nodes, and the evaluation of prognostic factors helps to identify its extent. The first described and most influential prognostic factors are ypT and ypN categories, which describe the size and extent of residual cancer. Later on, further prognostic factors are identified, such as histological subtype, grade, and presence of tumor propagation. Macroscopic and microscopic evaluation of resection specimens has an important role of the investigation of these above mentioned prognostic factors.^1^, ^4^ International guidelines aid the reproducibility of macroscopic and microscopic examinations and histopathology reporting.^5^, ^6^, ^7^ The latter is the gold standard of clinicopathological correlation on multidisciplinary sessions determining the additional possible treatment.

In Hungary, macroscopic examination and histopathological reporting are regularized by the 4th Breast Cancer Consensus Conference's guideline that was published in 2020.^5^ Furthermore, the macroscopic examination's process of cases received NAT requires substantial routine, because the identification of tumor bed and the measurement of the size of residual tumor may be challenging. Differences between regressive changes and residual cancer in the tumor bed require radiopathological correlation and, in certain cases, mammographic evaluation of a sample or even each individual slice of the tumor bed is necessary. Because of the complexity of macroscopic and microscopic processing of these specimens, these patients should be operated in breast surgery centers and surgical specimens should be examined by pathologists specialized in breast pathology.

Residual cancer burden (RCB) is a novel prognostic tool that takes the most important features of residual cancer into account. It was introduced by Symmans et al.^8^ in 2007, and was designed for survival prediction of patients who received NAT. In their study, the tumor size measured in two dimension, the cellularity, the percentage of ductal carcinoma in situ (DCIS), the number of metastatic (axillary) lymph nodes, and the largest diameter of metastatic deposit have been identified as independent prognostic factors having an impact on distant relapse‐free survival (DRFS).^8^ An online application was created that classifies breast cancer patients treated with NAT on the basis of recurrence‐free survival (RFS).^9^ The algorithm calculates a RCB score and a RCB class for each case. The first subgroup of RCB is pathologic complete regression (pCR) that signifies the absence of invasive cancer in the tumor bed and in the axillary lymph nodes. Categories RCB I, RCB II, and RCB III identify an increasing extent of residual breast cancer with decreasing expected RFS estimates.^9^

Concerning reproducibility, Peintinger et al.^10^ have demonstrated excellent agreement with Spearman's correlation. In keeping with their results, Naidoo et al.^11^ published similar results with Spearman's correlation.

It has to be mentioned that determination of the interclass correlation coefficient (ICC) is the latest method developed to evaluate the reproducibility of quantitative measurements made by different observers measuring the same quantity. ICC is a more natural measure of association than Pearson's correlation.

The aim of our study was to demonstrate our “good practice” regarding the macroscopic and microscopic evaluation of breast cancer specimens after NAT, and to examine the reproducibility of RCB with ICC method.

## METHODS

Patients treated with NAT at the Department of Oncotherapy and operated on in the Department of Surgery in the University of Szeged were included in our retrospective study.

Clinicopathologic data were collected from medical charts, namely age, gender, histological type, grade, hormone receptor status (estrogen receptor [ER], progesterone receptor [PR], HER‐2, and Ki‐67) of the previous core biopsy, type of NAT, type of surgery of the breast, and the axilla. The following data of the surgical specimen were collected from histopathology reports: histological type, ygrade, tumor regression (TR) and nodal regression (NR) categories, ypT, ypN, anatomic and prognostic stages, completeness of resection, vascular, lymphatic and perineural invasion, largest diameter of tumor, and number of positive lymph nodes.

**FIGURE 1:**
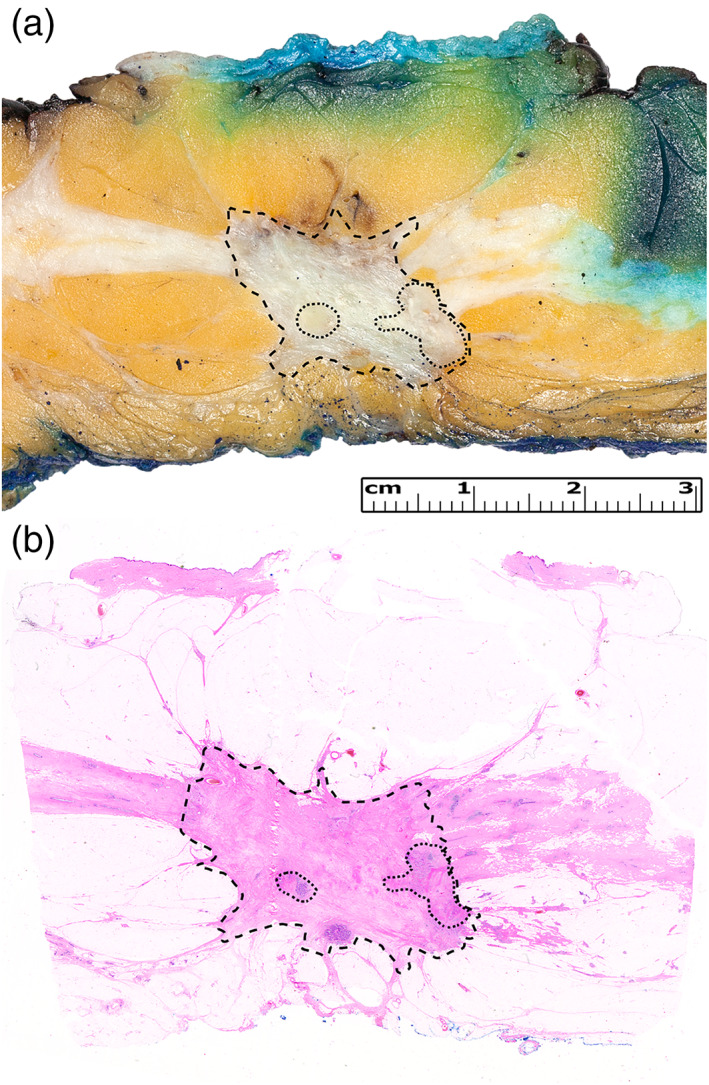
(a) Macroscopic image of a neoadjuvant therapy (NAT) treated mastectomy specimen that has been processed in coronal plane from lateral to medial resection margins. On the top, resected and intact skin is visible, while underneath an ill‐defined, flexibly firm, greyish tumour bed area is seen with surrounding fibrosis. On the bottom, blue ink is visible that was used to mark posterior resection margin. (b) From the exact slice, a macroblock was prepared and reflecting the tumour bed, besides the abnormal fibro‐connective tissue proliferation, caused by the NAT, residual invasive and in situ carcinoma was visible, as well. Tumour bed and residual invasive and in situ carcinoma are both outlined. The case was concluded as partial regression.

Gross examination is the key factor for the determination of RCB. Grossing of NAT breast cancer specimens in our department are handled by the regulations of the 4th Breast Cancer Consensus Conference's guideline, by breast cancer trained specialists, or specialist trainees.^5^ First and foremost, the most recent radiological examination's results are thoroughly read through by the dissection performing doctor, therefore, he or she has an elementary idea about the tumor's size, the localization, and the possibility of multifocality. After orientation of the specimen, the anterior resection margin is painted black and the posterior one is colored with blue stain. The specimen is cut in sagittal level, and each slice should have an utmost and general 4–5 mm thickness. Consequently, the localization of the tumor inside the specimen occurs with measuring all three dimensions and the distances from all resection margins (anterior, posterior, medial, lateral, superior, and inferior). From every tumor or tumor bed‐containing slice, a representative slide has to be made. If possible, the largest dimension of the tumor could be represented via macroblock technique. Finally, random quadrant slices are assembled, and dissection of the mamilla is performed. Figure 1 represents a NAT treated mastectomy specimen with partial regression. The specimen has been processed in coronal plane from lateral to medial resection margins, according to the 4th Breast Cancer Consensus Conference's guideline, and posterior resection margin is marked by blue stain.^5^ Underneath the resected and intact skin, a well‐defined grayish‐whitish tumor bed area is visible with surrounding fibrosis (Figure 1(a)). Histologically, the tumor bed was composed of abnormal fibro‐connective tissue proliferation caused by the NAT alongside with residual invasive and in situ carcinoma (Figure 1(b)).

At weekly oncoteam meetings, the evaluation of each case was based on a multidisciplinary consensus of radiological and clinicopathological correlation reflecting that the determination of the final tumor size is reliable.

Exclusion criteria included those cases that had the largest diameter in the mediolateral direction because of the mastectomy specimens’ sagittal manner of slicing. Furthermore, the cases that were too large to be measured on a normal or a macroslide were excluded as well.

Five pathologists with at least 1 year of breast pathology practice evaluated all cases independently, without being aware of the former histopathological results of the cases. Olympus BX43 and Nikon Alphaphot 2 microscopes were used for the evaluation. In every case, the two largest diameters of the tumor, the tumor cellularity, the percentage of DCIS, and the size of largest metastatic deposit were registered.

RCB points and classes have been assembled by using The University of Texas MD Anderson Cancer Center website (http://www3.mdanderson.org/app/medcalc/index.cfm?pagename=jsconvert3) and therefore, RCB points and subclasses were identified.^12^

Statistical analysis was carried out by SPSS Statistics V.22.0 software (IBM, SSPS 22.0). Our analytic method was ICC. Depending on the interpretation of each criterion's reproducibility two‐way random, single measures, and absolute tests were used based on the work of Koo et al.^13^ Significant reproducibility was set when the ICC was at least 0.9. ICC was chosen instead of κ statistics, and the former is used for continuous quantitative variables and the latter is for categorical variables.

This retrospective study was approved by the institutional ethical committee of the Albert Szent‐Györgyi Clinical Centre of the University of Szeged.

## RESULTS

**FIGURE 2:**
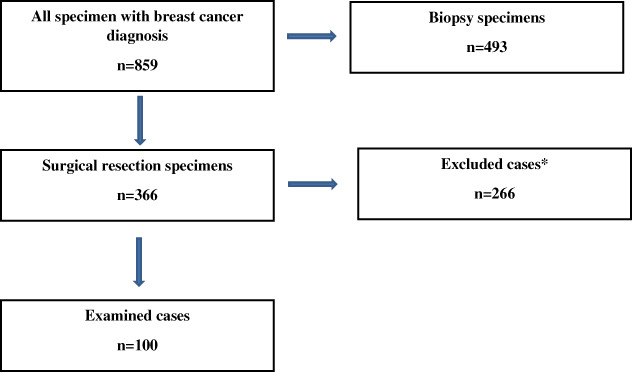
Flow chart of the patient selection process. Cases were collected between 2018–2021. *Exclusion occurred because of lack of neoadjuvant therapy (NAT) or if the largest diameter of the tumor was located in the mediolateral direction in the histological slides. Furthermore, the cases that were too large to be measured on a normal or a macroslide were excluded as well.

Figure 2 displays the patient selection and the numbers of excluded cases. The mean age of patients was 57 years (range, 26–88), and all of them were females. Table 1 summarizes the histological subtype, the grade, the molecular subtype of the examined tumors, based on the immunohistochemical evaluation of the core biopsy specimen, the clinical stage, and the oncological and surgical treatment. Almost all cases were primarily diagnosed as no special type (NST) carcinomas (*n* = 92). Two‐third of patients (*n* = 68) had grade 3 tumor. Most patients belonged to the best prognostic luminal A group, and one third of them had TNBC. Principally, patients (*n* = 74) received primary chemotherapy, and nearly all of them were given third generation medications (*n* = 70). Regarding clinical stage, almost half of the cases (*n* = 48) belonged to cT2N0M0 and cT2N1M0 categories. Despite NAT, 28% of patients (*n* = 28) underwent breast conserving surgery. Regarding axillar surgery, two‐third of patients had axillary block dissection. In few cases (*n* = 3), the axillar surgery occurred before the beginning of the NAT.

**TABLE 1:** Summary of the initial molecular subtype of all examined cases, oncological and surgical treatment

	No.	%
Histological subtype (core needle biopsy)		
NST carcinoma	92	92
Invasive lobular carcinoma	4	4
Invasive micropapillary carcinoma	1	1
Solid papillary carcinoma	1	1
Invasive mucinous carcinoma	1	1
In situ carcinoma^a^	1	1
Grade (core needle biopsy)		
1	6	6
2	26	26
3	68	68
Molecular subtypes (core needle biopsy)		
LumA (ER and/or PR+, HER2−)	38	38
LumB (ER and/or PR+, HER2−)	10	10
HER2 (ER−, PR−, HER2+)	19	19
TNBC (ER+, PR+, HER2+)	33	33
Clinical stage (core needle biopsy)		
Stage I	4	4
Stage II	38	38
Stage III	51	51
Stage IV	7	7
Type of NAT		
Primary chemotherapy	70	70
Primary endocrine therapy	14	14
Combined primary chemo‐ and endocrine therapy	3	3
Radiation therapy	3	3
Primary endocrine and radiation therapy	2	2
Primary chemo‐ and immunotherapy	1	1
No data	7	7
Type of surgery		
Breast conserving therapy	28	28
Mastectomy	72	72
Type of axillar surgery		
Sentinel lymph node resection	36	36
Axillary block dissection	61	61
Axillar surgery happened before initiation of NAT	3	3

^a^ In the histologically in situ carcinoma case, because of the radiological findings on tumor size raised suspicion for invasive malignancy, therefore, the lesion has been removed.

The surgical specimens’ main histological features are summarized in Table 2. Complete pCR was achieved in one‐third of cases. The most common histological subtype was NST carcinoma (*n* = 57). Others criteria included mucinous, micropapillary, and tubular carcinomas. There was no significant difference found between grades. One‐third of cases remained ypT2 after NAT. In the majority of cases, the axillar examination resulted in ypN0 category.

**TABLE 2:** Histopathological features of all surgical specimens

	No.	%
Histological diagnosis of surgical specimen		
pCR	29	29
DCIS	3	3
No special type carcinoma	57	57
Invasive lobular carcinoma	5	5
Others	6	6
ypT		
0	29	29
is	3	3
mic	0	0
1	27	27
2	30	30
3	9	9
ypN		
0	57	57
mi	2	2
1	28	28
2	7	7
3	2	2
Axillar surgery happened before initiation of NAT	3	3
No data	1	1
Number of metastatic lymph nodes		
0	57	57
1–5	29	29
6–10	3	3
>10	7	7
Axillar surgery happened before initiation of NAT	3	3
No data	1	1

Table 3 includes statistical results of ICC. Significant concordance was found in the two largest diameters of the tumor (coefficients, 0.984 and 0.973), the cellularity (coefficient, 0.970), and the largest metastatic deposit in lymph node (coefficient, 0.998). Although the amount of in situ carcinoma proved to be the least reproducible factor, it resulted in almost 90% of agreement (coefficient, 0.873). Regarding RCB points and classes, similar results were observed (coefficients, 0.989 and 0.960).

**TABLE 3:** Summary of the results of interclass correlation

Examined factor	Interclass correlation coefficient	95% confidence interval	*F*‐test value	*df*1	*df*2	*p*
Largest tumor diameter (D1)	0.984	0.977–0.989	63.22	70	280	<0.0001
Second largest tumor diameter (D2)	0.973	0.962–0.982	37.82	70	280	<0.0001
Cellularity	0.970	0.947–0.982	46.24	70	280	<0.0001
Amount of in situ carcinoma	0.873	0.819–0.914	7.85	70	280	<0.0001
Largest metastatic deposition	0.998	0.997–0.999	541.6	70	280	<0.0001
RCB point	0.989	0.983–0.992	94.38	69	276	<0.0001
RCP class	0.960	0.943–0.973	25.23	69	276	<0.0001

## CONCLUSIONS

Initiation of NAT revolutionized the therapy of locally advanced breast cancer.^1^ According to the results of recent publications, molecular subtype, ystage, ygrade, tumor infiltrating lymphocytes, and lymph node status has already been identified as prognostic factors of NAT treated breast cancer cases.^14^, ^15^, ^16^ Best prognosis has been associated with patients diagnosed with HER‐2 positive cancer or TNBC.^1^, ^2^, ^3^

RCB was developed by Symmans in 2007, and was designed as a survival prediction tool for NAT receiving patients. The algorithm creates RFS predictions based on the tumor size measured in two dimension, the cellularity, the percentage of DCIS, the number of metastatic (axillary) lymph nodes, and the largest diameter of metastatic deposit.^8^ Later, Symmans and colleagues^9^ evaluated the prognostic role of RCB, focusing on molecular subtypes and oncologic therapy, by using Kaplan–Meier analysis and log rank test. Continuous RCB index was observed in each prognostic category, which reflected the accuracy of RCB in prognosis estimation.

In our previous study, the prognostic impact of all currently available regression grading systems was evaluated, and in accordance with the results of Symmans and associates, RCB proved to be the most optimal prognostic system of all.^1^ Laas et al.^2^ performed a comparison study of RCB and Neo‐Bioscore, and although the results generally reflect better performance for Neo‐Bioscore, RCB proved to be better in the different breast cancer subtypes especially in luminal and TNBC cases.

Regarding the reproducibility of RCB, so far only two studies could be found via thorough literature search. Peintinger et al.'s^10^ and Naidoo et al.'s^11^ results have demonstrated excellent agreement between examiners with Spearman's correlation. In Peintinger et al.'s^10^ study, five pathologists evaluated 100 NAT cases. The concordance correlation coefficient concerning RCB score proved to be 0.795 and 0.704 regarding primary tumor bed size and 0.699 in fraction of invasive tumor size. Naidoo et al.'s^11^ study included 90 cases, examined by two expert pathologists. Overall concordance was solely examined regarding RCB scores and subgroups (0.9497 and 0.9145); however, the articles do not mention the other examined factors in more detail.^10^, ^11^ Table 4 assesses the results of these reproducibility examinations. Regardless of these recent studies using Spearman's correlation, it has to be emphasized that ICC is the latest method developed to evaluate the reproducibility of quantitative measurements made by different observers measuring the same quantity.

**TABLE 4:** Summary of the results of the current reproducibility examinations of RCB

Author	Year of publication	Journal	No. of examiners	No. of cases	Conclusion
Naidoo et al.^11^	2017	Histopathology	2	90	Good agreement (Spearman's correlation coefficient for RCB score = 0.9497; 95% CI = 0.9235–0.9671; *p* < 0.0001 and Spearman's correlation coefficient for RCB class = 0.9145; 95% CI = 0.8712–0.9437; *p* < 0.0001)
Peintinger et al.^10^	2015	Modern pathology	5	100	Good agreement (Spearman's correlation coefficient = 0.931 (95% confidence interval [CI] 0.908–0.949); κ coefficient: 0.583 (95% CI 0.539–0.626)

The aim of our study was to demonstrate our “good practice” regarding the macroscopic and microscopic evaluation of breast cancer specimens after NAT, and to examine the reproducibility of RCB with ICC method. Five pathologists with at least 1 year of breast pathology practice evaluated all cases independently, and registered the two largest diameters of the tumor, the tumor cellularity, the percentage of DCIS, and the size of largest metastatic deposit without being aware of the former histopathological results of the cases. RCB points and classes have been assembled by using the online available RCB calculator, therefore, RCB points, and subclasses were identified.

Significant concordance was found in the two largest diameters of the tumor (coefficients, 0.984 and 0.973), the cellularity (coefficient, 0.970), and the largest metastatic deposit in lymph node (coefficient, 0.998). Although the amount of in situ carcinoma proved to be the least reproducible factor, it resulted in almost 90% of agreement (coefficient, 0.873). Keeping with the results of previous publications, RCB points and classes have been shown to be reproducible prognostic parameters (coefficients, 0.989 and 0.960).

Strengths of our study are the good practice regarding the macroscopic evaluation of breast cancer specimens, the routine utilization of macroslides, the evaluation of the largest diameters in the antero‐posterior and supero‐inferior direction on macroslides, and the clinicoradiologic and pathologic correlation of all cases on multidisciplinary team. Furthermore, a newer and more accurate statistical analysis was performed. We would also like to highlight the fact, that currently there are only two former studies examining the reproducibility of RCB, both with similar results. In these studies, the reproducibility of each component is not specified, whereas our study highlights the fact that the percentage of DCIS is the most subjective and least reproducible parameter. The limitations of our research include the exclusion of tumors with the largest diameter in the mediolateral direction. In this setting, only macroscopic description can help determine tumor size and this evaluation was not reproducible.

Because of the NAT's affects, grossing and histological examination of these specimens remain a challenging task, therefore, it is recommended to be used by pathologists specialized in breast pathology. However, according to our results, significant agreement was observed between the examiners based on almost all RCB parameters, points, and classes reflecting the optimal reproducibility of RCB. Therefore, we recommend the use of the calculator in routine histopathological reports in NAT cases.

## AUTHOR CONTRIBUTIONS

Database – Anita Sejben.

Concept and design – Anita Sejben, Tamás Zombori.

Evaluation of histopathology slides – Anita Sejben, Fanni Hegedűs, Szintia Almási, Orsolya Oláh‐Németh, Tamás Zombori.

Consensus on near cut‐off staining cases – all authors.

Residual Cancer Burden calculation – Márton Berta.

Statistical analysis – Tamás Zombori, Anita Sejben.

Search and evaluation of references – Anita Sejben, Tamás Zombori.

Drafting the manuscript – Anita Sejben, Tamás Zombori.

Approval of final manuscript – all authors.

## CONFLICT OF INTEREST STATEMENT

The authors declare no conflict of interest.

## ETHICS APPROVAL STATEMENT

The Ethical Committee of the Albert Szent‐Györgyi Medical Center of the University of Szeged was consulted and approved this non‐interventional retrospective study. The institutional data safety manager also gave approval for this study not requiring patients' identity related data.

## Data Availability

The authors confirm that the data supporting the findings of this study are available within the article.
